# Gait Speed Maintenance Is Associated With Sensorimotor and Frontoparietal Network Connectivity Among Older Adults

**DOI:** 10.1093/geroni/igab046.2083

**Published:** 2021-12-17

**Authors:** Chun Liang Hsu, Brad Manor, Lewis Lipsitz

**Affiliations:** 1 Hinda and Arthur Marcus Institute for Aging Research, Harvard Medical School, Burnaby, British Columbia, Canada; 2 Hinda and Arthur Marcus Institute for Aging Research, Harvard Medical School, Boston, Massachusetts, United States; 3 Hebrew SeniorLife, Boston, Massachusetts, United States

## Abstract

Mobility impairment is a geriatric giant. Particularly, slow gait is associated with elevated risk for cognitive decline, disabilities and dementia. Gait is the product of complex neural network interactions and changes in their connectivity pattern may negatively impact gait speed. However, mechanistic neural correlates for gait speed maintenance and decline remained unclear. As such, the aim of this study is to investigate differences in neural network connectivity in older adults with and without gait speed decline over 24 months. This sub-analysis included 35 community-dwelling older adults age >70 years from the MOBILIZE Boston Study. Baseline assessments included four-meter gait speed test and resting-state fMRI. Gait speed was reassessed at a 24-month follow-up. Participants were stratified to “Maintainer” and “Decliner” groups based upon a cut-off of >0.05 m/s decline in gait speed from baseline to follow-up. A priori selected functional network included sensori-motor network (SMN) and frontoparietal network (FPN). Multivariate analysis of variance was performed to determine between group differences in network connectivity. Discriminant analysis was conducted to identify relative contribution of network connectivity to group classification. Between the 14 Maintainers and 21 Decliners (mean age 83.9 years), Maintainers were younger (p=0.088). After adjusting for age, Maintainers exhibited lower SMN premotor-precentral gyrus connectivity (p=0.023), greater FPN ventral visual-supramarginal gyrus connectivity (p=0.025), and trend level greater SMN-FPN cerebellum-occipital connectivity (p=0.053). Premotor-precentral gyrus connectivity showed greatest contribution to discriminant function. These preliminary findings suggest aberrant connectivity patterns of the SMN and FPN may be predictive of older adults’ ability to maintain gait speed.

